# Assessing Temporal Changes in Spatially-Varying Disparities in Tobacco Retailer Density across Ohio

**DOI:** 10.18061/ojph.v7i2.9993

**Published:** 2025-06-25

**Authors:** Rui Qiang, Peter F. Craigmile, Wendy Hyde, Abby Shores, Megan E. Roberts

**Affiliations:** 1 Department of Statistics, The Ohio State University, Columbus, OH; 2 Department of Mathematics and Statistics, Hunter College, City University of New York, New York, NY; 3 Department of Allied Health, Sport, and Wellness, Baldwin Wallace University, Berea, OH; 4 College of Public Health, The Ohio State University, Columbus, OH

**Keywords:** Tobacco retailer density, Tobacco control, Disparities, Equity, Policy, Tobacco retailer licensing

## Abstract

**Background:**

Place-based disparities in tobacco retailer density (TRD) are related to place-based disparities in tobacco use. This project aimed to assess the equity of changes in TRD disparities for various communities over the last 5 years. In addition, we sought to explore how changes varied as a function of local tobacco retailer licensing policies.

**Methods:**

In 2017 and 2022, we geocoded all tobacco retailers (including hookah cafés and vape shops) in Ohio and used census-derived information to categorize 3149 census tracts based on their demographic characteristics. With these data, we calculated cross-sectional TRD disparities, then estimated changes in TRD from 2017–2022. We also assessed tracts that had (vs had not) implemented tobacco retailer licensing. Analyses used negative binomial models adapted to account for spatial association across tracts and temporal dependence over years.

**Results:**

There was hardly any change in overall TRD over the 5-year period (1.77% decline). However, disparities were slightly attenuated for tracts with a high prevalence of Hispanic individuals, children, poverty, and African American individuals. The TRD did not decline for rural (vs suburban) areas; furthermore, rurality was one of the strongest predictors of TRD. In suburban and urban areas (where tobacco retailer licensing was most common), TRD declined more in high-poverty tracts that did (vs did not) have tobacco retailer licensing.

**Conclusion:**

Declines in TRD were greater for some communities than others. In particular, there was no indication that TRD is declining in rural areas of the state. Findings indicate the need for support and expansion of state and local-level tobacco control policies.

## INTRODUCTION

The term “tobacco retailers” refers to all types of stores that sell tobacco products; these can include gas stations, convenience stores, grocery stores, dollar stores, pharmacies, tobacco shops, vape shops, etc. Unfortunately, the locations of tobacco retailers are not uniformly distributed. Rather, there are disparities in tobacco retailer density (TRD), meaning that tobacco retailers are disproportionately located in systematically divested neighborhoods including low-income neighborhoods, neighborhoods with a high prevalence of racial or ethnic minority individuals, and rural areas.^1–6^ And these disparities in tobacco retailer density (TRD) are related to disparities in tobacco use.7 Such an association is to be expected: tobacco retailers are not only a major point of access to tobacco products but also a primary source of exposure to tobacco marketing.^8^ Consequently, living in neighborhoods with a high TRD has been associated with greater tobacco use and worse cessation outcomes.^7,9–12^ There have even been linear relationships found between degrees of disparity in TRD and degrees of disparity in tobacco use.^13^

Although a robust literature of cross-sectional data has documented these TRD disparities, it is important to recognize that the location of tobacco retailers is not static over time. Rather, thelocations of tobacco retailers are dynamic and impacted by numerous factors. For example, there was greater volatility in retailer closings and openings following the Great Recession of 2007–2009,^14^ and economic hardships associated with the COVID-19 pandemic impacted many retailer closures and turnover.^15–17^ Additionally, local-level tobacco control efforts targeting the retail environment are being adopted by many communities.^18^ Chief among these is tobacco retailer licensing, where a retailer is required to purchase a license to sell tobacco.^19^ The cost of the license, which typically must be renewed annually, can be a disincentive for selling tobacco.^20^ The tobacco retailer licensing also provides funds and infrastructure for local retail enforcement including compliance checks and penalizing or suspending retailers for repeated sales violations (eg, sales to underage youth).^21^ Thus, the number and distribution of tobacco retailers can change substantially over time.^14^

But what has been the impact of these changes in tobacco retailers for TRD disparities? There are many gaps in our understanding of this topic. Unfortunately, some data indicate disparities in tobacco use are rising.^22^ One of the only studies assessing changes in TRD found that, from 2000–2017, poverty-based disparities in TRD reduced while racial and ethnic-based disparities remained unchanged.^23^ Whether these trends have continued in recent years remains unknown. Also unknown are how trends change over time for rural (vs urban) areas, and across the intersection of community characteristics (eg, low-income racial minority neighborhoods vs high-income racial minority neighborhoods). Finally, little is known about how tobacco retailer licensing impacts changes in TRD disparities.

This project’s objective was to assess recent longitudinal changes in TRD disparities that have historically been observed cross-sectionally at the neighborhood level: disparities based on neighborhood income, racial and ethnic composition, and rurality. In exploratory analyses, we also examined how these changes varied as a function of tobacco retailer licensing. Analyses were conducted for the state of Ohio, as this is a large state (over 44 000 square miles and a population of over 11.7 million) with a varied sociodemographic profile and good representation of our groups of interest. Further, Ohio was unique in having no tobacco retailer licensing at baseline (2017) but several jurisdictions implementing tobacco retailer licensing over the course of a 5-year period.

## METHODS

### Measures

Tobacco Retailers. In 2017, and again in 2022, the names and addresses of all retailers with active state cigarette licenses (gas stations, grocery stores, tobacco shops, etc) were obtained from Ohio’s county auditor offices. To collect information on hookah cafés and vape shops that did not have a state cigarette license, we employed methods described by Kates et al^24^ for searching internet directories. Our final list contained 11 458 tobacco retailers in 2017 and 11 341 in 2022 (including hookah cafés and vape shops, which together comprised 3% of retailers in 2017 and 4% in 2022). We geocoded the longitude-latitude coordinates corresponding to the retailer addresses using the tidygeocoder^25^ R package.

Sociodemographic Characteristics. For all Ohio census tracts (“tracts”), we obtained information about race/ethnicity, poverty, age, and population size from the 2016 and 2022 American Community Survey (ACS) 5-year estimates. The 2016 ACS values were used as covariates in modeling the tobacco retailer counts in 2017; the 2022 ACS values were used as covariates in modeling the retailer counts in 2022. For this paper, we were particularly interested in identifying trends for historically divested census tracts, characterized by poverty, race/ethnicity, and rurality. Cutoffs distinguishing “high” and “low” groups were selected a priori and justified elsewhere.^26^ Tracts were coded for high (vs low) prevalence of African Americans [or Hispanics] if ≥15% of the population was African American [or Hispanic]. Tracts were coded for high (vs low) prevalence of young people if ≥25% of the population was under age 18. Finally, tracts were coded for high (vs low) prevalence of poverty if >15.4% of the population was below the poverty level (15.4% was the state poverty level in the 2016 ACS). To aid in the comparison over the 2 time periods, we also used 15.4% to define a high (vs low) prevalence of poverty in 2022. To determine whether a tract was urban, rural, or suburban, we used the National Center for Health Statistics’ 2013 Urban-Rural Classification Scheme for Counties.^27^ A level 1 county was coded as “urban,” levels 2 and 3 were coded as “suburban,” and levels 4, 5, and 6 were coded as “rural.”

The TIGER shapefiles defining tracts in Ohio came from the US Census Bureau.^28^ Our procedure for configuring sociodemographic variables across 2 timepoints on a single set of 2021 census tracts is described in the Appendix. Following our established methodology to guard against low retailer counts,^26^ we restricted our analyses to tracts with a minimum population of 500 people (17 tracts had populations of <500 people, 15 had no population). Two more tracts were removed for having missing poverty values. Our final analysis had data for 3149 tracts.

Tobacco Retailer Licensing. Although Ohio already has a state-level retailer license for cigarettes, more comprehensive local tobacco retailer licensing had begun appearing in the state. In addition to including all types of tobacco products beyond cigarettes (eg, e-cigarettes, cigars, hookah), the local tobacco retailer licensing required annual license fees and provided stronger infrastructure for enforcement, such as unannounced compliance checks for underage sales, with penalties for violations (including fines and suspended or revoked licenses). We compiled a list of all localities in Ohio that enacted a tobacco retailer licensing policy before 2022; none of these tobacco retailer licensing policies were enacted before 2017 (our baseline period). This list comprised 13 Ohio cities, including those within the highest population counties: Cuyahoga, Franklin, and Hamilton (Table 1). We obtained shapefiles of the cities of Columbus and Cincinnati from the Centers for Disease Control and Prevention.^29^ For smaller cities, we manually traced city boundaries using Google Maps and calculated which 2021 tracts were contained within, or had at least a 50% overlap with, each of these cities.

### Statistical Analyses

Analyses were carried out using R.^30^ Analyses began with descriptive statistics to map and characterize tracts and TRD at both timepoints. The TRD was calculated as the number of retailers per 1000 people in a tract. Using our common set of tracts, we determined the median TRD and percentage change in median TRD across high vs low levels of our sociodemographic characteristics.

Any instance where median TRD was greater for divested, compared to nondivested, neighborhoods (eg, tracts with high vs low prevalence of poverty) was considered a TRD disparity. And any instances where the percent change in median TRD was greater for divested, compared to nondivested, neighborhoods was considered an equitable decline in TRD.

Next, we fit a statistical model to understand the relationship between TRD and sociodemographic variables in 2016 and 2022, while accounting for possible spatiotemporal dependencies. We used a marginal modeling approach, which specifies a model for the mean, variance, and correlation. The model for the log mean TRD accounts for the effect of sociodemographic variables that could be different over years, as well as the urban/suburban/rural status of the tract. The variance of a negative binomial model allows for overdispersion in the response^26^ (ie, extra variance relative to what we could observe in a Poisson model). For the correlation model, we assumed a conditional autoregressive (CAR) model over tracts and an autoregressive (AR) model over time. The Appendix provides further details on the statistical model and fitting methodology.

Finally, to explore the impact of local tobacco retailer licensing on TRD in 2022, we added an indicator variable to our statistical model that indicated whether tobacco retailer licensing was enacted within that tract (yes or no). We then compared TRD change predicted from the model for different combinations of sociodemographic variables. For this exploration, we fixed the age group to be a low prevalence of children. Recognizing similar patterns across high-prevalence African American tracts and high-prevalence Hispanic tracts, we compared low-African American/low-Hispanic tracts to high-African American/high-Hispanic tracts.

## RESULTS

### Tobacco Retailer Density (TRD) 2017 and 2022

**Figure 1.**
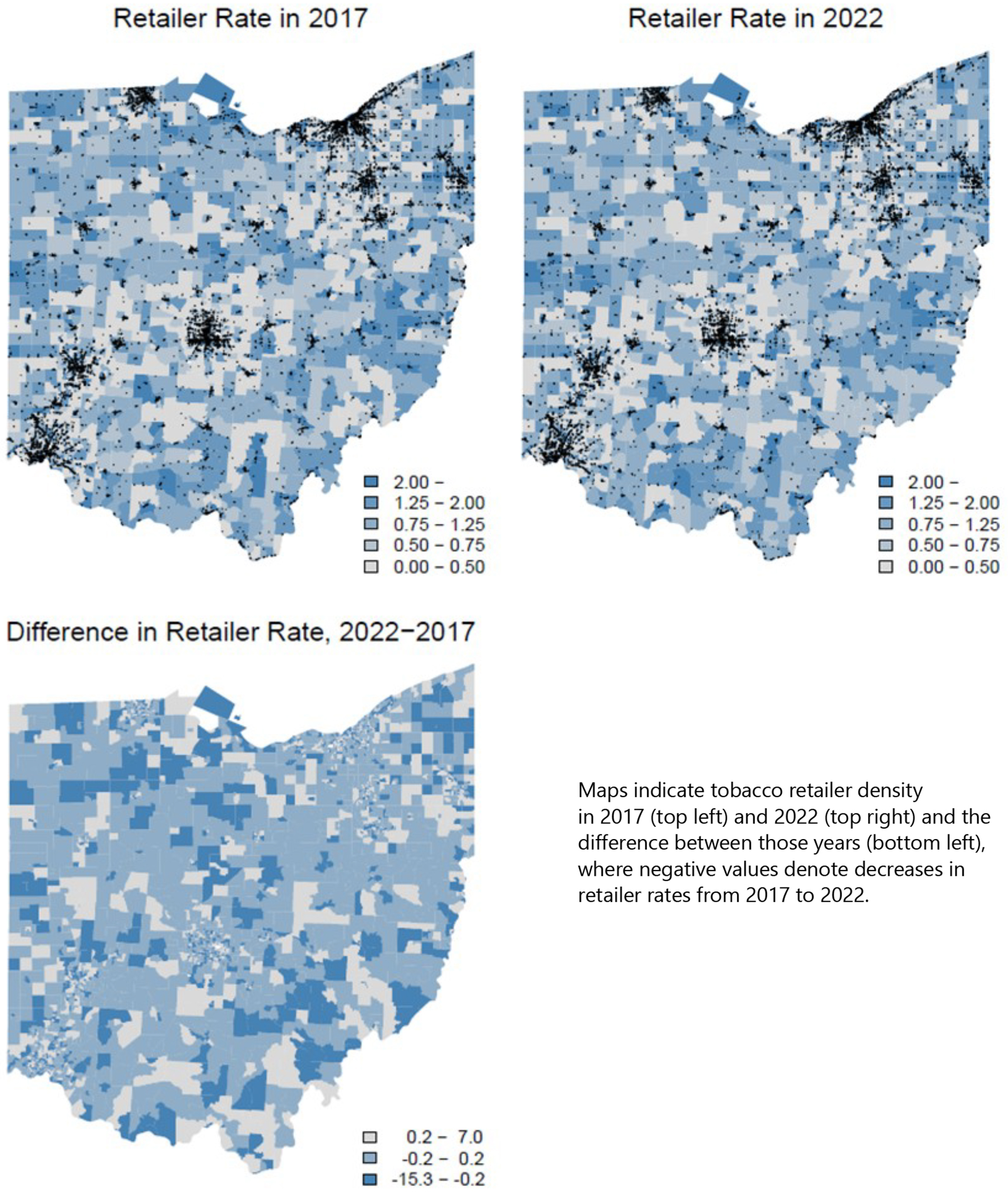
Ohio Tobacco Retailer Density Maps at Census Tract Level Figure notes: Black points indicate retailer locations. Top row: Darker colors indicate greater tobacco retailer density, measured as number of retailers per 1000 people. Bottom row: Darker colors indicate greater increase in tobacco retailer density over the 5-year period.

For the state of Ohio, there was a 1.77% statewide reduction in TRD between 2017 and 2022. However, there was substantial variation across tracts in both the direction and magnitude of TRD change over this 5-year period (Figure 1). We found 22.1% of tracts experienced an increase in TRD from 2017–2022; among these, the mean increase was 0.50 retailers per thousand people. Another 24.5% of tracts experienced a decrease in TRD from 2017–2022; among these, the mean decrease was 0.66 retailers per thousand people. Thus, across tracts, the decrease slightly outweighed the increase.

### Tobacco Retailer Density (TRD) Disparities—Descriptive Statistics for Cross-Sectional and 2017–2022 Changes

The distribution of ACS-based sociodemographic characteristics changed somewhat in Ohio over our period of observation (Table 2). As compared to 2017, the prevalence of tracts in 2022 classified as “high prevalence African American,” “high prevalence under 18,” and “high poverty” decreased, and the prevalence of tracts classified as “high prevalence Hispanic” increased. Median TRD decreased from 2017–2022 for tracts classified as both high- and low-prevalence African American, with a greater decrease in high-prevalence tracts (a 2.5% decrease vs 1.3% decrease, respectively; Table 2). Median TRD decreased by 14.7% for tracts classified as high-prevalence Hispanic and 2.5% for tracts classified as low-prevalence. For tracts with a higher prevalence of people aged under 18 years, the decrease of 6.6% was higher than the decrease for tracts with a lower prevalence (2.2%). In terms of poverty, TRD decreased 2.2% for high-poverty tracts, but increased by 2.1% for low-poverty tracts. Finally, we observed a decrease for urban tracts and suburban tracts (0.8% and 3.2%, respectively) but a slight increase in TRD of 0.3% for rural tracts.

### Multivariable Models of TRD Disparities–2017 and 2022

After applying Wald tests to simplify the model, the only interaction term we retained in our model was the interaction between the prevalence of children (ie, people under age 18) and poverty (Table 3). The final model (Table 3, Model 1) indicated that, at both timepoints, there was significantly greater TRD in tracts with a high (vs low) prevalence of African Americans (exp (0.138)=1.15 times as many retailers in 2017; exp(0.101)=1.11 times as many in 2022). There was also significantly greater TRD in tracts with a high (vs low) prevalence of Hispanic individuals (1.25 times in 2017; 1.19 times in 2022). There was no significant difference in TRD between suburban and urban tracts in 2017; however, by 2022, there was significantly greater TRD in suburban vs urban tracts (1.09 times as many). At both timepoints, there was significantly greater TRD in rural vs urban tracts (1.30 times as many in 2017 and 1.36 times in 2022).

In both 2017 and 2022, there was significantly lower TRD in tracts with a high (vs low) prevalence of people under 18 and greater TRD in tracts with high (vs low) poverty. The children × poverty interaction indicated that the association between TRD and poverty was particularly pronounced where there was a high prevalence of children.

### Tobacco Retailer Licensing

In terms of the impact of tobacco retailer licensing, we observed that tracts with tobacco retailer licensing (13 cities, or 430 tracts) showed a greater decrease in TRD (6.6%) vs those tracts that did not have tobacco retailer licensing (2.8%; Table 2). In our second marginal model (Table 3, Model 2), which included tobacco retailer licensing as a factor, the estimated term for the tobacco retailer licensing policy effect was not statistically significant. Overall, patterns between our first model (without the tobacco retailer licensing term) and our second model (with the tobacco retailer licensing term) were very similar; the only major difference was that the effect of suburban tracts was no longer significant in the second model.

**Figure 2.**
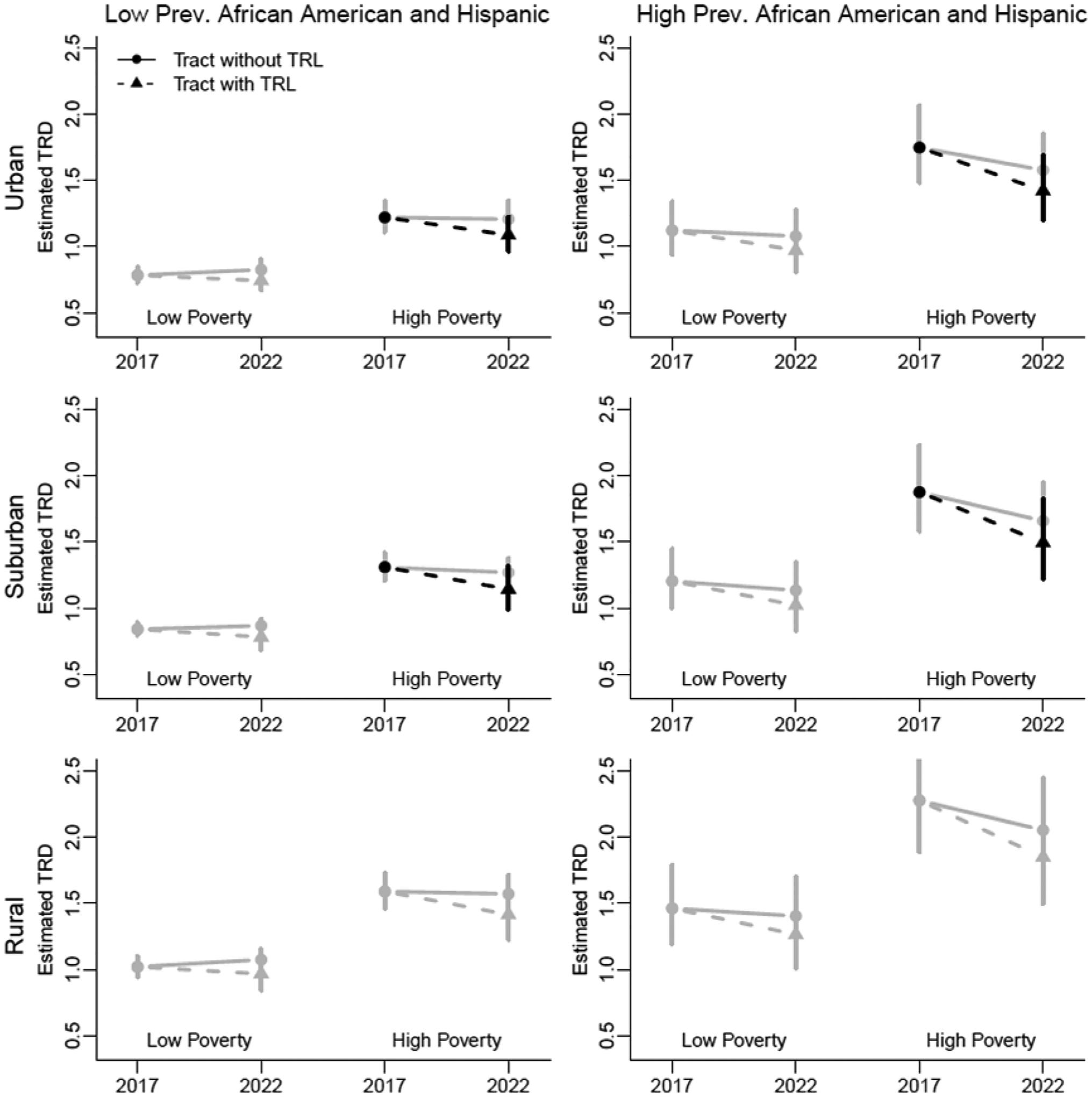
Estimated Tobacco Retailer Density for Census Tracts Grouped by Sociodemographic Variables and Year Figure notes: Dashed lines indicate tracts with a tobacco retailer license policy. Solid lines indicate tracts without a tobacco retailer license policy. Lines colored black indicate a significant difference between 2017 and 2022. Gray lines are not significant. Error bars indicate 95% confidence intervals.

Regardless of racial or ethnic composition, high-poverty urban and suburban tracts with tobacco retailer licensing experienced a significant decrease in TRD (Figure 2). While there is a suggestion that the TRD may have decreased for other communities with tobacco retailer licensing, the decrease was not statistically significant.

## DISCUSSION

This paper observed a 1.77% decline between 2017–2022 in TRD for Ohio overall. However, the rate of TRD decline was greater for some communities than others. Specifically, TRD declined the most for tracts with a high prevalence of Hispanic individuals and a high prevalence of children (ie, population under the age of 18). There were also some modest declines for tracts with a high prevalence of poverty and a high prevalence of African American individuals. Thus, the degree of TRD disparities was attenuated for these communities, but not eliminated; indeed, our marginal model indicates TRD was still associated with the poverty, race and ethnicity, age, and rurality of an area’s residents in 2022. These present findings somewhat align with previous US data, which found poverty-based TRD disparities declined over time, but racial and ethnic-based disparities remained unchanged.23 Whether any of the equitable declines in Ohio constitute meaningful change for the communities is difficult to determine. But there is evidence that even moderate differences in TRD (eg, 0 vs >5 retailers in an area) are associated with differences in smoking prevalence.^31^

Whereas TRD declined in suburban areas, there was no indication that TRD was declining equitably for rural areas. These findings underscore how progress toward equity does not always advance at the same rate for all populations. It is encouraging to see TRD disparities reduced for areas with high poverty and a high prevalence of racial or ethnic minority individuals. However, it is concerning that no such declines occurred for rural areas. In fact, our modeling indicates rurality is one of the strongest predictors of TRD. There are many potential reasons for this continuing rural disparity. As discussed below, support and capacity for local tobacco control policy likely plays a role. Another potential factor is the predatory nature of certain tobacco retailer chains. For example, discount stores (or “dollar stores”) are more highly concentrated in rural areas^32^ and are one of the only types of tobacco retailers whose numbers continue to increase.^14^

This study also observed some evidence of an equitable decline in TRD in locations that implemented tobacco retailer licensing. The TRD significantly declined in high-poverty urban and suburban areas with (vs without) tobacco retailer licensing. Such outcomes support statements by tobacco control advocates that tobacco retailer licensing could be an equitable strategy for reducing TRD.^19,21^ The outcomes also align with research emerging from other areas of the United States33,34 pointing to real-world equitable effects of tobacco retailer licensing. This promising finding arrives at a difficult time for Ohio, as state legislators approved state preemption of all local tobacco policies in early 2024,^35^ effectively erasing the benefits of local tobacco retailer licensing. Even more recently, public health champions won a lawsuit arguing this preemption law violated the state constitution, meaning local policy is again allowed—but only for the (mostly urban) localities that were part of the lawsuit.^36^ Consequently, we may see the public health benefits of tobacco retailer licensing continue to grow for these primarily urban communities.

It is noteworthy that nearly all tobacco retailer licensings enacted in Ohio were in urban or suburban areas. Thus, it is likely we did not detect an effect of tobacco retailer licensing in rural areas because we have no statistical power to do so. Statistical power may also explain why we did not detect an overall effect of tobacco retailer licensing in our marginal models. This policy-based disparity in tobacco retailer licensing may have also contributed to our finding, discussed above, that TRD disparities did not decline for rural tracts. Unfortunately, rural areas are often left behind in policy innovation, as they frequently lack the capacity needed to successfully introduce tobacco control policies, contributing to disparities in tobacco use.^37,38^

Limitations to the present study should be acknowledged. Our analysis used dichotomized covariates and there may be nonlinear models that describe the relationship between TRD and the sociodemographic covariates when dichotomization is not used. Our data came from just one US state, and additional research will be needed to determine whether the present outcomes generalize to other states or countries. Our data also captured a time period made distinctive by the COVID-19 pandemic; while critical to capture, the trends and patterns observed may not extend to future years. Our investigation with tobacco retailer licensing should also be interpreted with caution, given the somewhat low prevalence of tobacco retailer licensing investigated (13 cities, comprising 13.7% of the state’s tracts).

## PUBLIC HEALTH IMPLICATIONS

The present findings indicate little overall change in Ohio’s TRD over a 5-year period. Depending on the type of community, there were some equitable declines in TRD, which is encouraging. However, our modeling indicates the TRD of an area is still significantly associated with the poverty, race and ethnicity, age, and rurality of its residents. Based on these findings, and knowing that disparities in TRD are associated with disparities in tobacco use,^7^ it is likely that tobacco-related health concerns will continue to disproportionately impact high-poverty individuals, racial and ethnic minority individuals, and rural individuals in Ohio.

Findings from this study can inform other localities considering retailer-based policies. To precipitate more drastic change in TRD, tobacco retailer licensing could be supplemented with licensing-law strategies, such as restricting retailers from being close to schools or capping the number of retailers allowed in a county,^39^ which will likely yield equitable effects.^33,40,41^ Policy makers may also wish to consider even stronger licensing approaches, such as age-restricted location policies. Traditional approaches to addressing the retail environment, such as enforcement of minimum-age-of-sale laws, also require continued focus. Throughout these efforts, particular attention should be paid to policy implementation in rural areas, as these are among the communities most disadvantaged by TRD, while simultaneously the least served by retailer-based tobacco control. Rather than leaving the decision to pass tobacco retailer licensing to local officials, state-level policies may be necessary to ensure equitable, comprehensive coverage.

## Supplementary Material

1

## Figures and Tables

**Table 1.** Ohio Cities That Passed Local Tobacco Retailer Licensing Policy between 2017 and 2022

City	County	Tobacco retailer density (TRD) in 2017 (per 1000 people)^a^	Population in 2017 (thousands)^b^
Brook Park	Cuyahoga	0.85	18.8
Brooklyn	Cuyahoga	1.27	11.0
Cleveland Heights	Cuyahoga	0.71	45.0
Euclid	Cuyahoga	0.90	47.9
Lakewood	Cuyahoga	1.11	46.8
Maple Heights	Cuyahoga	1.54	22.7
Moreland Hills	Cuyahoga	0.25	4.0
Newburgh Heights	Cuyahoga	1.26	7.1
University Heights	Cuyahoga	0.30	13.3
Columbus	Franklin	0.93	887.7
Dublin	Franklin	0.45	44.7
Cincinnati	Hamilton	1.16	304.7
Norwood	Hamilton	1.38	19.6

a Tobacco Retailer Density (TRD) is calculated over all census tracts containing the city.

b Population was calculated as the aggregate population over all census tracts containing the city.

Notes: Includes county, tobacco retailer density (TRD), and population. Corresponds to 430 census tracts (13.7% of all tracts in state).

The TRD over all other Ohio census tracts (ie, those not included in the table) is 0.99 per thousand people in 2017.

**Table 2.** Sociodemographic Characteristics and Tobacco Retailer Density (TRD) in 2017 and 2022, by census tracts in Ohio

Characteristic	Prevalence (% Census Tracts)			Median Tobacco Retailer Density (per 1000 people)		
	2017	2022	% change	2017	2022	% change
African American						
High prevalence^a^	26.7	26.6	**−0.5**	1.18	1.15	**−2.5**
Low prevalence	73.3	73.4	0.2	0.92	0.91	**−1.3**
Hispanic						
High prevalence^b^	4.1	4.9	17.7	1.55	1.32	**−14.7**
Low prevalence	95.9	95.1	**−0.8**	0.97	0.95	**−2.5**
Under 18 population						
High prevalence^c^	31.7	29.4	**−7.5**	0.95	0.88	**−6.6**
Low prevalence	68.3	70.6	3.4	1.02	1.00	**−2.2**
Poverty						
High prevalence^d^	42.9	38.0	**−11.4**	1.32	1.29	**−2.2**
Low prevalence	57.1	62.0	8.6	0.80	0.82	2.1
Neighborhood type^e^						
Urban	31.0	31.0	N/A	0.93	0.92	**−0.8**
Suburban	45.1	45.1	N/A	0.95	0.92	**−3.2**
Rural	23.9	23.9	N/A	1.12	1.13	0.3
Tobacco retailer licensing^f^						
Yes	0.0	13.7	N/A	0.98	0.91	**−6.6**
No	100.0	86.3	N/A	1.00	0.97	**−2.8**

a Tracts where 15% or more of the population is African American.

b Tracts where 15% or more of the population is Hispanic.

c Tracts where 25% or more of the population is under age 18.

d Tracts where more than 15.4% of the population is below the poverty level (15.4% is the state average for Ohio at baseline).

e Classification of urban, rural, and suburban is derived from the 2013 National Center for Health Statistics Urban-Rural Classification Scheme for Counties. Thus, the prevalence cannot change between 2017 and 2022.

f Tracts of cities in Ohio which passed a local tobacco retailer license ordinance between 2017 and 2022.

N/A = Not applicable. Change scores were not calculated.

Note: Sociodemographic data were drawn from the American Community Survey (ACS) in 2016 (paired with 2017 retailer data) and 2022 (paired with the 2022 data). The median tract population of 3575 in 2022 was slightly higher than the median tract population of 3535 in 2017 (the total population in Ohio increased by approximately 88 000 from 2017 to 2022).

Numbers in **BOLD** indicate a decrease from 2017 to 2022.

**Table 3.** Parameter Estimates (and standard errors) from Two Marginal Models Relating 2017 and 2022 Tobacco Retailer Density (TRD) to Sociodemographic Variables, while accounting for Spatiotemporal Dependence

Factor	Model coefficient (standard error)	
	2017	2022
**Model 1**		
Intercept	**− 0.244 (0.040)**	**−0.233 (0.039)**
High prevalence of African American	**0.138 (0.045)**	**0.101 (0.045)**
High prevalence of Hispanic	**0.221 (0.080)**	**0.175 (0.074)**
Neighborhood type		
Suburban vs Urban	0.070 (0.041)	**0.092 (0.041)**
Rural vs Urban	**0.264 (0.050)**	**0.306 (0.050)**
High prevalence of children	**−0.325 (0.050)**	**−0.355 (0.047)**
High prevalence of poverty	**0.443 (0.042)**	**0.376 (0.042)**
Poverty × children interaction	**0.165 (0.069)**	**0.248 (0.070)**
**Model 2: Tobacco retailer licensing term added**		
Intercept	**−0.244 (0.040)**	**−0.191 (0.045)**
High prevalence of African American	**0.138 (0.045)**	**0.106 (0.045)**
High prevalence of Hispanic	**0.221 (0.080)**	**0.161 (0.074)**
Neighborhood type:		
Suburban vs Urban	0.070 (0.041)	0.049 (0.048)
Rural vs Urban	**0.264 (0.050)**	**0.263 (0.055)**
High prevalence of children	**−0.325 (0.050)**	**−0.356 (0.047)**
High prevalence of poverty	**0.443 (0.042)**	**0.380 (0.042)**
Poverty × children interaction	**0.165 (0.069)**	**0.251 (0.070)**
Tobacco retailer licensing	-	−0.104 (0.060)

Note: **BOLD** font indicates effects are significantly different from zero, with significance level 0.05.
