# Supplementary material for: Assessing Temporal Changes in Spatially-Varying Disparities in Tobacco Retailer Density across Ohio
Source: Ohio J Public Health. Author manuscript; Available in PMC 2026 Feb 5. (PMC12872171; doi:10.18061/ojph.v7i2.9993)
Supplement: 1 [file NIHMS2139601-supplement-1.pdf]

APPENDIX

This Appendix describes how we define the temporally-varying sociodemographic variables on a common set of (2021) census tracts. We then provide details on the spatiotemporal statistical model that we assume for establishment counts over space and time. This generalizes the spatial model of Adibe et al[1] to spatiotemporal models. We also give an estimate of the covariance of the regression parameters using a sandwich estimator. See Figure S2.

S1 Procedure for configuring sociodemographic variables across 2 timepoints on a single set of census tracts
The shapefile for 2022 was not available when our tobacco retailer data were collected; therefore, our analysis is based on the 2021 shapefile. From 2017–2021, the tracts in Ohio changed, and the number of tracts increased from 2952 to 3168. Our analysis used 2021 tract configurations. To obtain a common set of sociodemographic variables on the same spatial scale over the 2 time points, we mapped the 2017 American Community Survey (ACS) demographic variables to the 2021 tracts by comparing the area of overlap in the 2017 and 2021 tracts. To calculate the 2017 population in each of the 2021 tracts, we re-weighted the 2017 populations by the proportion of areas of the 2017 tracts that overlapped with the 2021 tracts. For all other sociodemographic variables, we defined the 2017 ACS values for each 2021 tract as being the value found in the 2017 tract that had the greatest overlapping area with the 2017 tract. This process generated 2017 and 2022 ACS sociodemographic values defined on a common set of (2021) tracts.

S2 Defining the spatiotemporal model
Suppose that $i = 1, \dots, m$ indexes the m census tracts in Ohio, and let t denote the time index (in this application t = 1 denotes 2017 and t = 2 denotes 2022, but the model can allow for more than 2 time points). Let Y_{it} denote the number of establishment counts in census tract i and year t with P_{it} denoting the population of tract i in thousands for year t. Let $\boldsymbol{x}_{it}$ be a vector of covariates for each census tract i and time point t of length p_t, and $\boldsymbol{\beta}_t$ denote regression coefficients for each time point t. In our marginal model we assume that Y_{it} are spatially and temporally correlated with mean

$$\mu_{it} = \mathrm{E}(Y_{it}) = P_{it} \exp(\boldsymbol{x}_{it}^T \boldsymbol{\beta}_t), \tag{S1}$$

variance

$$V_t(\mu_{it}) = var(Y_{it}) = \frac{\sigma_t^2}{1-\phi^2}\left[\mu_{it} + \frac{\mu_{it}^2}{\theta_t}\right], \tag{S2}$$

and covariance

$$cov(Y_{it}, Y_{i't'}) = \sqrt{V_t(\mu_{it}) V_{t'}(\mu_{i't'})} R_{ii'} \, \phi^{|t-t'|}.$$

Here $\theta_t > 0$ is an overdispersion parameter that can vary in time, $\sigma_t^2 > 0$ is a variance parameter that can also vary in time, ϕ is a temporal dependence parameter that lies between −1 and 1, and $R_{ii'}$ is the (i, i') element of a $m \times m$ spatial correlation matrix $\boldsymbol{R}$ that corresponds to assuming a conditional autoregressive (CAR) spatial model (eg Banerjee et al[2]) across the m census tracts. We assume that $\boldsymbol{R}$ is defined by

$$\boldsymbol{R} = (\boldsymbol{D} - \alpha \boldsymbol{W})^{-1}, \tag{S3}$$

where $\boldsymbol{W}$ is a $m \times m$ spatial proximity matrix with (i, i') element equal to one if tract i and tract i' share a border, and zero otherwise. The diagonal elements of $\boldsymbol{W}$ are assumed to be zero. The $m \times m$ matrix $\boldsymbol{D}$ is a diagonal matrix with ith diagonal element equal to the number of census tracts that share a border with census tract i. In (S3), the parameter α denotes a spatial dependence parameter that lies between −1 and 1 and does not vary with time.
We use a generalized estimating equation (GEE) methodology to fit our model. We first fit negative binomial generalized linear models to the establishment counts across the m census tracts for each year t: for each time point t we fit a generalized linear model assuming (S1) and (S2), assuming independence over the different census tracts. We then perform statistical inference on the regression parameters $\boldsymbol{\beta}_t$ over time indexes t using a sandwich estimator that uses the spatiotemporal correlations assumed in (S3).
In terms of model building, starting with the covariates and interactions, we used Wald tests to simplify the model, leaving terms that were jointly significantly different from zero while accounting for the spatiotemporal dependence.

S3 Estimating the covariance matrix for the regression parameters
Let $\boldsymbol{Y}_t = (Y_{1t}, \dots, Y_{mt})^T$ denote the vector of establishment counts for time point t and $\boldsymbol{X}_t$ denote the $m \times p_t$ design matrix with ith row equal to the covariate vector $\boldsymbol{x}_{it}$ for census tract i at time point t. Let $\boldsymbol{G}_t$ be an $m \times p_t$ matrix with (i,j) element $\mu_{it}[\boldsymbol{x}_{it}]_j$, and let $\boldsymbol{J}_t = \boldsymbol{G}_t^T \boldsymbol{V}_t^{-1} \boldsymbol{G}_t$ where $\boldsymbol{V}_t = \mathrm{diag}(V_t(\mu_{it}): i = 1, \dots, m)$ is the $m \times m$ working covariance matrix assuming independence over space for each time point t. Then, the sandwich estimator for the covariance of the estimated regression parameter $\widehat{\boldsymbol{\beta}}_t$ at time point t is

$$\mathrm{cov}(\widehat{\boldsymbol{\beta}}_t) = \boldsymbol{J}_t^{-1} \boldsymbol{G}_t^T \boldsymbol{V}_t^{-1} \mathrm{cov}(\boldsymbol{Y}_t) \boldsymbol{V}_t^{-1} \boldsymbol{G}_t \boldsymbol{J}_t^{-1}$$

and the covariance between regression parameters at different time points t and t' is

$$\mathrm{cov}(\widehat{\boldsymbol{\beta}}_t, \widehat{\boldsymbol{\beta}}_{t'}) = \boldsymbol{J}_t^{-1} \boldsymbol{G}_t^T \boldsymbol{V}_t^{-1} \mathrm{cov}(\boldsymbol{Y}_t, \boldsymbol{Y}_{t'}) \boldsymbol{V}_{t'}^{-1} \boldsymbol{G}_{t'} \boldsymbol{J}_{t'}^{-1}.$$

The spatial and temporal dependence parameters α and ϕ are estimated from the Pearson residuals for all census tracts and time points using maximum likelihood (ML). With these estimates, our estimated covariance of the estimated regression parameter $\widehat{\boldsymbol{\beta}}_t$ at time point t is

$$\widehat{\text{cov}}(\hat{\boldsymbol{\beta}}_t) = \boldsymbol{J}_t^{-1}\boldsymbol{B}_t^T\left[\frac{\hat{\sigma}_t^2}{1-\hat{\phi}^2}\right](\boldsymbol{D}-\hat{a}\boldsymbol{W})^{-1}\boldsymbol{B}_t\boldsymbol{J}_t^{-1}$$

with the estimated covariance between the parameters at 2 different time points *t* and *t'* being

$$\widehat{\text{cov}}(\hat{\boldsymbol{\beta}}_t, \hat{\boldsymbol{\beta}}_{t'}) = \boldsymbol{J}_t^{-1}\boldsymbol{B}_t^T\left[\frac{\hat{\phi}\,\hat{\sigma}_t\,\hat{\sigma}_{t'}}{1-\hat{\phi}^2}\right](\boldsymbol{D}-\hat{a}\boldsymbol{W})^{-1}\boldsymbol{B}_{t'}\boldsymbol{J}_{t'}^{-1},$$

where $B_t = \text{diag}\left(\frac{\hat{\mu}_{it}}{\sqrt{V_t(\hat{\mu}_{it})}} : i = 1, \dots, m\right)\boldsymbol{X}_t$, for each time point *t.*

S4 Census tracts affected by tobacco retailer licensing policies between 2017 and 2022

Figure S1 displays a map of Ohio indicating the census tracts in blue affected by the enactment of tobacco retailer licensing policies between 2017 and 2022.

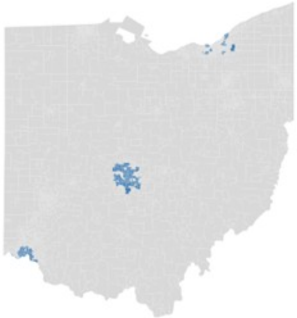

Figure S1 Ohio tobacco retailer licensing policies

S5 Tobacco Retailer Density (TRD) Ratios

Table S1 tabulates TRD ratios from 2 marginal models relating 2017 and 2022 TRD to sociodemographic variables, while accounting for spatiotemporal dependence.

For example, in Model 1 we estimate that in Ohio in 2017 the TRD density is 1.25 times higher for census tracts with a high prevalence of Hispanic vs census tracts with a low prevalence of Hispanic. A 95% confidence interval for this factor is between 1.07 and 1.46.

Table S1 The TRD ratios from 2 marginal models relating 2017 and 2022 TRD to sociodemographic variables, while accounting for spatiotemporal dependence. The numbers in parentheses are 95% confidence intervals for the ratios.

Factor	TRD Ratio (95% CI)	
	2017	2022
Model 1		
High prevalence of African American	**1.15 (1.05,1.25)**	**1.11 (1.01,1.21)**
High prevalence of Hispanic	**1.25 (1.07,1.46)**	**1.19 (1.03,1.38)**
Neighborhood type:		
Suburban vs Urban	1.07 (0.99,1.16)	**1.10 (1.01,1.19)**
Rural vs Urban	**1.30 (1.18,1.44)**	**1.36 (1.23,1.5)**
High prevalence of children	**0.72 (0.66,0.80)**	**0.07 (0.64,0.77)**
High prevalence of poverty	**1.56 (1.43,1.69)**	**1.46 (1.34,1.58)**
Poverty × children interaction	**1.18 (1.03,1.35)**	**1.28 (1.12,1.47)**
Model 2		
High prevalence of African American	**1.15 (1.05,1.25)**	**1.11 (1.02,1.21)**
High prevalence of Hispanic	**1.25 (1.07,1.46)**	**1.17 (1.02,1.36)**
Neighborhood type:		
Suburban vs Urban	1.07 (0.99,1.16)	1.05 (0.96,1.15)
Rural vs Urban	**1.30 (1.18,1.44)**	**1.30 (1.17,1.45)**
High prevalence of children	**0.72 (0.66,0.80)**	**0.70 (0.64,0.77)**
High prevalence of poverty	**1.56 (1.43,1.69)**	**1.46 (1.34,1.58)**
Poverty × children interaction	**1.18 (1.03,1.35)**	**1.29 (1.12,1.47)**
Tobacco retailer licensing	–	0.90 (0.80,1.01)

Bold font indicates effects are significantly different from 0.

S6 Local indicators of spatial association (LISA)

Using the sfweights R package (https://github.com/JosiahParry/sfweight), we ran a LISA analysis (Anselin[3]) using the local Moran's I statistic calculated for the log TRD for each year (2017 and 2022), using the same spatial neighborhood structure as we used in the spatial model. This version of the analysis classifies census tracts into 4 categories:

1. HH: high values surrounded by high values;
2. HL: high values nearby other low values;
3. LH: low values nearby other high values;
4. LL: low values nearby other low values.

Plots of the categories, by tract, for each year are shown in Figure S2. To investigate general trends, Table S2 shows a percentage breakdown of the categories jointly over the 2 years. Figure S2 and TableS2 suggest that for both years, high log TRD values surrounded by high log TRD values (HH) is the most common situation in both 2017 (33.1% of the time) and 2022 (33.2% of the time), and that this category tends to occur in urban, suburban, and rural areas. Low log TRD values nearby other low log TRD values (LL) is less common (23.4% of the time in 2017 and 22.6% of the time in 2022). Figure S2 and further calculation indicate that this category is less likely in rural areas.

While a test of association rejects the null hypothesis of independence between the categories in 2017 and 2022, with a p value close to zero, Figure S2 and Table S2 provide no persuasive evidence that the distribution of these categories have changed greatly over these years. The clusters of categories differ slightly, but a general pattern of change is not consistent from 2017 to 2022.

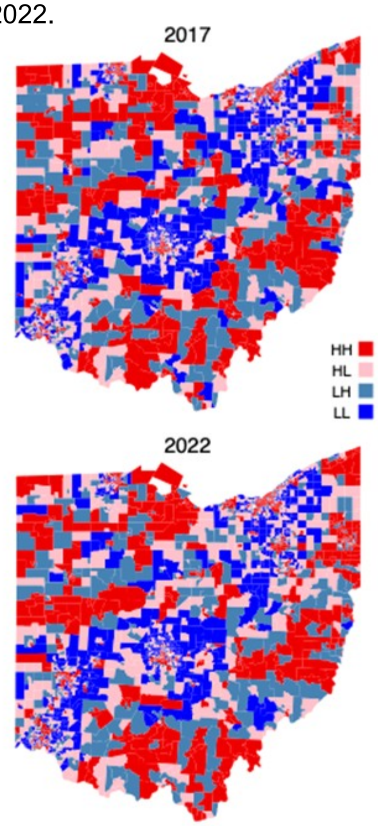

Figure S2: A map of Ohio in 2017 and 2022 indicating the clustering of log retailer rates for each census tract, as determined by calculating LISA. See text for further detail.

Table S2: A percentage breakdown of the LISA categories broken down over the 2 years, 2017 and 2022.

		2022			
		HH	HL	LH	LL
2017	HH	27.0	3.1	2.5	0.5
	HL	3.1	18.8	0.3	2.8
	LH	2.5	0.3	14.2	1.5
	LL	0.6	3.1	1.9	17.8

S7 Assessing the impact of retailer enforcement

To evaluate the possible role of retailer enforcement in our models for relating TRD to sociodemographic variables, we obtained data from the US Food and Drug Administration (FDA) on compliance check inspections of brick-and-mortar tobacco product retailers (downloaded from https://timp-ccid.fda.gov/). We pulled data from all FDA led inspections in Ohio during the year 2017, and again during the year 2022. There were 5251 inspections in 2017 and 3005 inspections in 2022. By county in Ohio, the number of inspections ranged from 1 to 175 in 2017, and from 0 to 974 in 2022.

For each census tract we calculated the number of inspections in 2017 per thousand people in the county that contains each census tract. We repeated the calculation for the number of inspections in 2022 per thousand people. There was no evidence of a linear relationship between these 2 covariates and the observed log retailers rates (we observed correlations with the observed log retailer rate of 0.051 for the 2017 inspections variable, and 0.046 for the 2022 inspections variable). Regardless, we added these 2 variables to Model 2 from the main article (Model 2 in-

cludes both the sociodemographic variables and a tobacco retailer licensing term.) A summary of the model in show in Table S3. This table illustrates that neither inspection variable was significant in our statistical model. Further, the estimated coefficients and associated standard errors hardly changed for the sociodemographic and tobacco retailer variables, indicating that when using these measures of retailer enforcement, there was no impact upon our findings.

Table S3: Parameter estimates from a marginal model relating 2017 and 2022 TRD to sociodemographic variables, while accounting for spatiotemporal dependence. This model includes covariates that measure the rate of inspections in 2017 and 2022, as well as a tobacco retailer licensing term in 2022. The numbers in parentheses are standard errors.

Factor	Model coefficient (standard error) 2017	2022
Model 3: Inspections in 2017 and 2022 and tobacco retailer licensing added		
Intercept	**-0.244 (0.040)**	**-0.191 (0.045)**
High prevalence of African American	**0.138 (0.045)**	**0.106 (0.045)**
High prevalence of Hispanic	**0.224 (0.080)**	**0.163 (0.074)**
Neighborhood type:		
Suburban vs Urban	0.059 (0.043)	0.036 (0.049)
Rural vs Urban	**0.246 (0.053)**	**0.252 (0.056)**
High prevalence of children	**-0.324 (0.050)**	**-0.352 (0.047)**
High prevalence of poverty	**0.443 (0.042)**	**0.380 (0.042)**
Poverty × children interaction	**0.163 (0.069)**	**0.246 (0.070)**
Tobacco retailer licensing		-0.104 (0.060)
Inspection rate	0.057 (0.056)	0.100 (0.089)

Bold font indicates effects are significantly different from 0.

References

1. Adibe C, Craigmile PF, Onnen N, Schwartz E, Roberts ME. The relationship between tobacco retailer density and neighborhood demographics in Ohio. *Ohio J Public Health*. 2019;2(1). https://doi.org/10.18061/ojph.v2i1.9036
2. Banerjee S, Carlin BP, Gelfand AE. *Hierarchical Modeling and Analysis for Spatial Data*. 2nd ed. Chapman and Hall/ CRC, 2014. https://doi.org/10.1201/b17115
3. Anselin L. Local indicators of spatial association — LISA. *Geogr Anal*. 1995;27:93-115. https://doi.org/10.1111/j.1538-4632.1995.tb00338.x
